# Effects of *Lactobacillus plantarum* PS128 on Depressive Symptoms and Sleep Quality in Self-Reported Insomniacs: A Randomized, Double-Blind, Placebo-Controlled Pilot Trial

**DOI:** 10.3390/nu13082820

**Published:** 2021-08-17

**Authors:** Yu-Ting Ho, Ying-Chieh Tsai, Terry B. J. Kuo, Cheryl C. H. Yang

**Affiliations:** 1 Institute of Brain Science, National Yang Ming Chiao Tung University, Taipei 11211, Taiwan; mascottofu@gmail.com (Y.-T.H.); tbjkuo@ym.edu.tw (T.B.J.K.); 2 Sleep Research Center, National Yang Ming Chiao Tung University, Taipei 11211, Taiwan; 3 Institute of Biochemistry and Molecular Biology, National Yang Ming Chiao Tung University, Taipei 11211, Taiwan; tsaiyc@ym.edu.tw; 4 Brain Research Center, National Yang Ming Chiao Tung University, Taipei 11211, Taiwan; 5 Department of Education and Research, Taipei City Hospital, Taipei 103212, Taiwan; 6 Clinical Research Center, Taoyuan Psychiatric Center Ministry of Health and Welfare, Taoyuan 33058, Taiwan

**Keywords:** *Lactobacillus plantarum* PS128, insomnia, depression, anxiety, heart rate variability

## Abstract

Recent animal studies have supported that *Lactobacillus plantarum* PS128 (PS128) can reduce the severity of anxiety and depression. However, previous studies did not focus on the sleep quality and mood of humans. This study determines whether PS128 reduces the severity of anxiety and depressive symptoms, regulates autonomic nervous system function, and improves sleep quality. Forty participants between 20 and 40 years of age with self-reported insomnia were randomly assigned to two groups, a PS128 group and a placebo group, in a double-blind trial. Participants took two capsules of either PS128 or a placebo after dinner for 30 days. Study measures included subjective depressive symptoms, anxiety and sleep questionnaires, and miniature-polysomnography recordings at baseline and on the 15th and 30th days of taking capsules. Overall, all outcomes were comparable between the two groups at baseline and within the 30-day period, yet some differences were still found. Compared to the control group, the PS128 group showed significant decreases in Beck Depression Inventory-II scores, fatigue levels, brainwave activity, and awakenings during the deep sleep stage. Their improved depressive symptoms were related to changes in brain waves and sleep maintenance. These findings suggest that daily administration of PS128 may lead to a decrease in depressive symptoms, fatigue level, cortical excitation, and an improvement in sleep quality during the deep sleep stage. Daily consumption of PS128 as a dietary supplement may improve the depressive symptoms and sleep quality of insomniacs, although further investigation is warranted.

## 1. Introduction

The microbiota–gut–brain axis is a bidirectional link involving the central nervous system, the enteric nervous system, and the gut microbiota. Accumulated evidence has demonstrated the importance of the gut microbiota in maintaining gastrointestinal homeostasis, boosting immune system, promoting nutrient absorption, etc. [1]. It also has become a potential therapeutic target for psychiatric disorders, such as depression [2], Parkinson’s disease [3], and dementia [4]. The effect of microbiota on the brain occurs through three main pathways of the microbiota–gut–brain axis: hormones and neurotransmitters, the immune system, and the vagus nerve [5]. One possible approach to actively modulate the gut microbiota to improve health is through probiotics, which are defined as “live microorganisms which when administered in adequate amounts confer a health benefit on the host” [6]. In addition, certain probiotics identified as “psychobiotics” can regulate the microbiota–gut–brain axis and bring health benefits to patients with mental illness, which holds promise for the treatment of insomnia [7].

Insomnia affects daily activities by causing inattention and drowsiness. The prevalence of insomnia is estimated to be 10−60% of the population [8]. Up to 40% of insomniacs also suffer from psychiatric disorders, with depression and anxiety being the most common [9]. Depression is more than just sadness, but a lack of energy and markedly diminished interest [10]. As for anxiety, it is “an emotion characterized by feelings of tension, worried thoughts and physical changes like increased blood pressure [11].” Other than that, most insomnia diagnoses are based on patient-reported symptoms. The diagnostic criteria in the Diagnostic and Statistical Manual of Mental Disorders, 5th Edition (DSM-5) for insomnia disorders includes at least one of the following [12]: difficulty initiating sleep, difficulty in maintaining sleep, and early morning awakening with inability to return to sleep. Chronic insomnia is diagnosed when a patient has any of these conditions for at least three nights a week for three months or longer.

Current treatments for insomnia are either risky or only gradually effective. Often taken for their rapid effect, sleeping pills are prescribed to millions of patients every year [12], and none of these sedative-hypnotic drugs are risk free [13]. Common negative side effects include addiction, fatigue, and long-term alterations in brainwave activity [14]. Cognitive behavioral therapy for insomnia (CBT-I) is regarded as the non-medical treatment that best improves sleep quality and is the first-line treatment for chronic insomnia, with an efficacy that is unquestionable [15]. However, patients consider it too time-consuming, complicated, and not much more effective than medication [16], and the premature dropout rate of CBT-I is approximately 40% [17]. In addition, untreated insomnia may carry a significant economic cost, besides complications such as depression, anxiety, and cardiovascular disease [9]. Therefore, finding a safe and convenient treatment would provide substantial benefits. Although the exact cause of insomnia is unknown, many studies support the hyper-arousal theory [18], which states that insomnia results from a dysfunctional hypothalamic–pituitary–adrenal (HPA) axis [19]. The HPA axis plays a central role in the stress response, and is also involved in digestion, endocrine activity, immunity, and mood. Evidence shows that during sleep, insomniacs have increased high-frequency electroencephalogram (EEG) activity, adrenocorticotropic hormone (which stimulates cortisol release) levels, heart rates, and autonomic nervous system (ANS) activity compared to healthy people [20,21]. Moreover, neurotransmitters also seem to play a role in insomnia. Insomniacs often have an imbalance of neurotransmitters that drive the sleep-wake cycle [22]. These include those that induce sleep, such as gamma-aminobutyric acid (GABA), adenosine, and melatonin, and those that promote waking, such as noradrenaline, serotonin, acetylcholine, orexin, and dopamine.

Sleep is closely related to the gastrointestinal microbiota. Factors such as sleep deprivation [23], working night shifts [24], and circadian disorders [25] can change the structure of gastrointestinal microbiota and circadian gene expression. Some research has shown that supplemental probiotics can enhance sleep quality or relieve stress [26,27,28,29], but most of these studies only measured sleep quality using subjective questionnaires. In addition to subjective measures, we used miniature polysomnography (PSG), which provides the most direct objective assessment of sleep architecture. *Lactobacillus plantarum* PS128 (PS128^TM^) is a psychobiotic strain developed in Taiwan [30]. Preclinical studies have shown that PS128 can increase levels of dopamine and serotonin in the brain to ameliorate psychiatric symptoms in rodents [31,32]. Therefore, we hypothesized that PS128 can enhance sleep quality by ameliorating mood and reducing cortical excitation in self-reported insomniacs. Therefore, we conducted this study to determine (i) whether there is a relationship between PS128 and depressive symptoms or anxiety, (ii) whether PS128 could regulate the ANS by reducing the sympathetic nervous system and increasing the parasympathetic nervous system during sleep, (iii) whether PS128 could improve sleep quality by increasing sleep efficiency, maintaining sleep duration, and decreasing sleep latency, and (iv) whether the change in sleep quality is related to mood.

## 2. Materials and Methods

### 2.1. Experiment Design

This is a randomized, double-blind, parallel, placebo-controlled pilot trial. During the screening period, sex, age, body mass index (BMI), blood pressure (BP), health habits, and medical history of all participants were recorded. Participants completed a sleep log for one week to record their daily schedules and sleep habits. They wore an electrocardiogram (ECG) patch all day to objectively measure their sleep-wake cycle, and an oximeter during sleep to exclude sleep apnea. After the first week, all participants were evaluated by miniature-PSG for two nights, to objectively assess their sleep quality. Participants came to the sleep laboratory at night to affix the miniature-PSG before returning home to sleep. For the first night, the miniature-PSG was worn only for adaptation. Baseline data were collected during the second night. A day was selected in which participants were evaluated by PSG at midday (three hours) to exclude other potential sleep problems.

**Figure 1:**
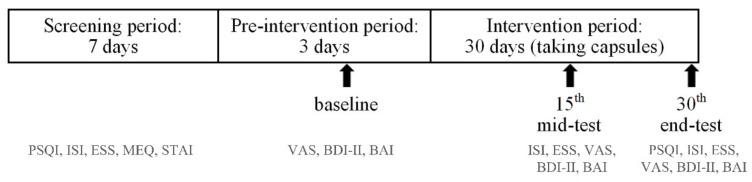
Study schedule. Participants underwent miniature-polysomnography at baseline, mid-test, and end-test. PSQI, Pittsburgh Sleep Quality Index; ISI, Insomnia Severity Index; ESS, Epworth Sleepiness Scale; BDI-II, Beck Depression Inventory-II; BAI, Beck Anxiety Inventory; STAI, State-Trait Anxiety Inventory State; MEQ, Morningness-Eveningness Questionnaire; VAS, Visual Analogue Scale.

After the above evaluations, participants started taking two capsules of either PS128 or placebo after dinner for 30 days. On the 15th and 30th days after they started taking the capsules, participants were evaluated by miniature-PSG as mid-test and end-test data. On the day of the miniature-PSG recording, the visual analogue scale (VAS) [33] was used to assess the relaxation level, fatigue level, and sleep quality. The Pittsburgh Sleep Quality Index (PSQI) [34] and Insomnia Severity Index (ISI) [35] were used to evaluate subjective sleep quality and insomnia severity. Daytime sleepiness was measured using the Epworth Sleepiness Scale (ESS) [36], depression was assessed using the Beck Depression Inventory-II (BDI-II) [37], anxiety levels were assessed using the Beck Anxiety Inventory (BAI) [38] and the State-Trait Anxiety Index (STAI), and circadian rhythm was assessed using the Morningness-Eveningness Questionnaire (MEQ) [39]. The study schedule is shown in Figure 1.

All participants were instructed to continue maintaining their sleep log and to avoid all the other probiotic products or antibiotics during the entire study period. They were also instructed not to consume caffeine on the day of baseline measurement, and days 15 and 30. This study was approved by the Institutional Review Board of National Yang Ming Chiao Tung University, and registered through ClinicalTrials.gov with identifier NCT04592276. Informed consent was obtained from all participants prior to enrolment.

### 2.2. Participants

Self-reported insomniacs were included if they met the following inclusion criteria: (i) aged 20–40 years; (ii) 18.5 < BMI < 25; (iii) systolic BP < 140 mmHg and diastolic BP < 90 mmHg; (iv) PSQI > 5, ISI >13; and (v) met the DSM-5 criteria for chronic primary insomnia. The fifth recruiting criterion of self-reported insomniacs was based on previous studies [40,41,42]. At the first screening, we confirmed that the patients’ duration of sleep problems was not shorter than three months, and that they had not sought any medical treatments. [39,40,41]. Potential participants were excluded if they had (i) used other probiotic products within the last two weeks; (ii) antibiotic treatment within the last month; (iii) taken sleep medication within the last two months, or were on long-term use; (iv) reported tobacco, alcohol, caffeine, or drug addiction; (v) lactic acid bacteria allergy; (vi) cancer, cardiovascular disease, psychiatric illness, kidney disease, diabetes mellitus, or other sleep disorders; (vii) inflammatory bowel disease; (viii) hepatobiliary or gastrointestinal tract surgery; or (ix) worked night shifts.

### 2.3. Test Capsules

PS128 and placebo capsules were provided by Bened Biomedical Co. Ltd. (Taipei, Taiwan). Each participant received a jar containing 60 capsules. Each PS128 capsule contained 3 × 10^10^ colony-forming units with microcrystalline cellulose. The placebo capsules contained only microcrystalline cellulose. Each PS128 or placebo capsule weighed 425 ± 25 mg and had the same appearance. Both were distributed and stored at 4 °C. The participants were asked to keep the jar in the refrigerator. Each jar had different numbers, and the numbers were used to determine each participant’s allocation group once the trial ended. Compliance was confirmed by the unused capsules returned by the participants.

### 2.4. Measurement and Analysis of Miniature-PSG

Miniature-PSG (TD1, Taiwan Telemedicine Device Company) [43,44] can detect four-channel electrophysiological signals (electrooculogram, EOG; electromyogram, EMG; EEG; ECG). It measures 5.2 × 3.1 × 1.2 cm and weighs 1 g. According to the International 10–20 system, EEG electrodes are placed at the C3-A2. The EOG electrodes were placed 1cm below and lateral to the left outer canthus, and 1 cm above and lateral to the right outer canthus. A pair of EMG electrodes was placed side by side on the chin. The ECG electrode was placed on the V5 site of the chest. Respectively, the EEG, EOG, EMG, and ECG were amplified to 2000, 1000, 1000, and 250, and filtered at 0.34–53 Hz, 0.034–53 Hz, 16–113 Hz, and 1.6–113 Hz. These signals were synchronously digitized with a resolution of 12 bit with different sampling rates.

The raw data were converted into the European Data Format, and then analyzed with RemLogic 2.0 software (Embla System Inc., Broomfield, CO, USA) by a qualified sleep technician who was blinded to participant allocation. The sleep timeline was divided into 30 s epochs. The sleep staging guidelines were based on the American Academy of Sleep Medicine 2017 manual, which scores sleep into four stages: rapid eye movement (REM) and non-rapid eye movement stages 1 (N1), N2, and N3. Sleep parameters recorded were total sleep time (TST), sleep onset latency (SOL), wake time after sleep onset (WASO), sleep efficiency (SE), number of awakenings, and arousal index.

### 2.5. EEG Power Spectral Analysis

The EEG spectral analysis during sleep was analyzed as previously described [43,44]. The EEG signals were calculated and truncated into continuous 64 s time segments with 50% overlapping. The power density of the spectral components was estimated with a non-parametric fast Fourier transform. First, the baseline drift of the signal was eliminated to prevent low-frequency interference. Then, a Hamming window was applied to each time segment to minimize the leakage effect. After that, we corrected the resulting power spectrum for attenuation caused by sampling and applying the Hamming window. The power density spectrum was quantified by integration, and the power of each frequency band of EEG power was as follows: delta (0.5–4 Hz), theta (4–8 Hz), alpha (8–13 Hz), and beta (13–32 Hz). Another EEG index used was the normalized EEG power (e.g., beta%), which was calculated as the power at a given frequency band divided by the total power (0.5–32 Hz). For further spectrograms of EEG, please refer to the previous study conducted by our laboratory [43].

### 2.6. Heart Rate Variability (HRV) Analysis

HRV is measured by variations in the R-R interval, between successive peaks of the QRS complex in the ECG wave. Based on fast Fourier transform, time-domain (R-R interval) transfer to frequency domain resulting in total power (TP, 0.0−0.4 Hz), low frequency (LF, 0.04−0.15 Hz), high frequency (HF, 0.15−0.4 Hz), and normalized LF (LF%) were obtained. LF% and HF represent an index of sympathetic activity and parasympathetic activity, respectively [44,45].

### 2.7. Statistical Analysis

The primary outcomes of the present study were the differences in sleep EEG before and after the consumption of probiotics, compared to the placebo group. The secondary outcomes included BDI-II, BAI, PSQI, ISI, ESS, HRV, VAS, and STAI. All statistical analyses were conducted using SPSS Statistics for Windows, version 18 (SPSS Inc., Chicago, IL, USA). *p*-values less than 0.05 were considered statistically significant. Background characteristics were compared between groups using the *t*-test. EEG data, HRV data, and questionnaires were compared between study groups using generalized estimating equations (GEE). Sleep structure, brainwaves, and HRV data were adjusted for age and sex. Subjective questionnaires and objective parameters were analyzed by one-way repeated-measures analysis of variance (ANOVA) within groups. PSQI data were assessed using the paired *t*-test within groups. The differences within groups at days 15 and 30 were compared to baseline or zero by 95% interval analysis. The correlations between the △BDI-II score and objective parameters were analyzed using Spearman’s rank correlation.

## 3. Results

**Figure 2:**
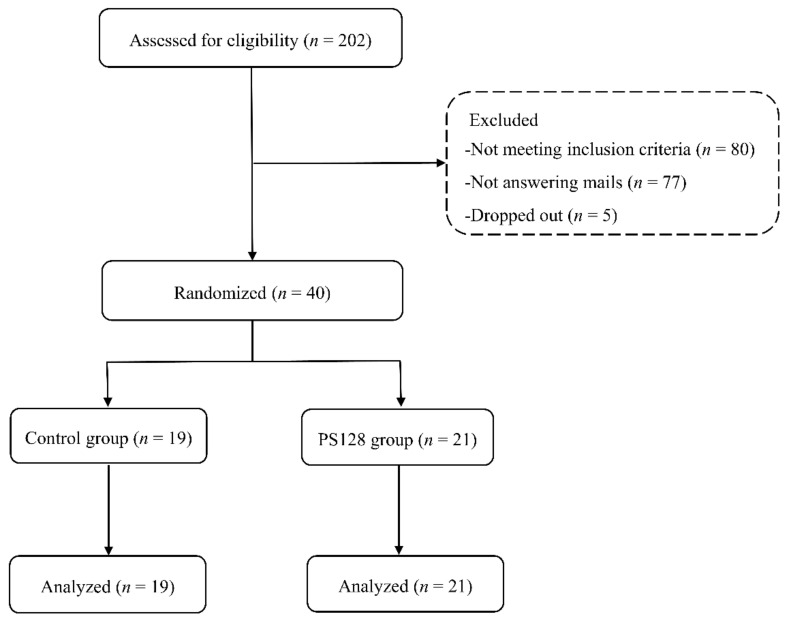
Participant flow: 202 potential participants contacted us. We evaluated participant eligibility using questionnaires, interviews, and sleep dairies. In the end, 40 participants were included.

Participants were recruited from October 2018 to December 2019. Of the 202 participants who were initially interested in the study, 80 were excluded for not meeting all the inclusion criteria. In total, 77 stopped replying, and five dropped out before taking any capsules. The remaining 40 participants were randomly allocated to either the PS128 or placebo group; 21 participants in the PS128 group and 19 participants in the placebo group were included in the final analysis. The participant flow diagram is shown in Figure 2. Both study groups were further divided into subgroups of insomniac (Inso) and misperception (Mis). This was based on the participants’ SOL or WASO, with 30 min as a cut-off point. Within the placebo group, 9 were identified as Inso and 10 as Mis. Within the PS128 group, 12 were identified as Inso and 9 as Mis. Blinding was confirmed at the end of the study by asking each participant to guess the treatment received. Additionally, the majority of the tested capsules had been used; the participants’ compliance rate was more than 98%, and no harmful events related to test capsule intake were reported.

The background characteristics of the 40 eligible participants are shown in Table 1. The study participants were predominantly female, and BDI-II and BAI scores showed that they experienced depression and anxiety at mild to normal levels of severity. The participants’ circadian rhythms were in between morning and evening types, based on the MEQ. None of the parameters that were assessed differed between the PS128 and placebo groups. However, in the subgroups, the BMI of Mis participants was significantly lower in the PS128 group than in the placebo group (*p* < 0.05), and the PSQI scores of Inso participants were significantly higher in the PS128 group than in the control group (*p* < 0.05).

### 3.1. Effects of PS128 on Subjective Parameters

The PSQI (both groups: *p* < 0.01, day 30 versus baseline) and ISI scores (control group: day 15, *p* < 0.05, day 30 < 0.01 versus baseline; PS128 group: day 15 and day 30 < 0.01 versus baseline) of both the PS128 and placebo groups decreased significantly, but showed no significant difference between groups. The ESS scores of the control group significantly decreased at days 15 and 30 (day 15 < 0.05, day 30 < 0.01) compared to baseline. The PS128 group appeared to be decreased at day 30 (*p* = 0.064) compared to baseline. The fatigue level on VAS before sleep in the control group showed a significant increase at day 30.

**Figure 3:**
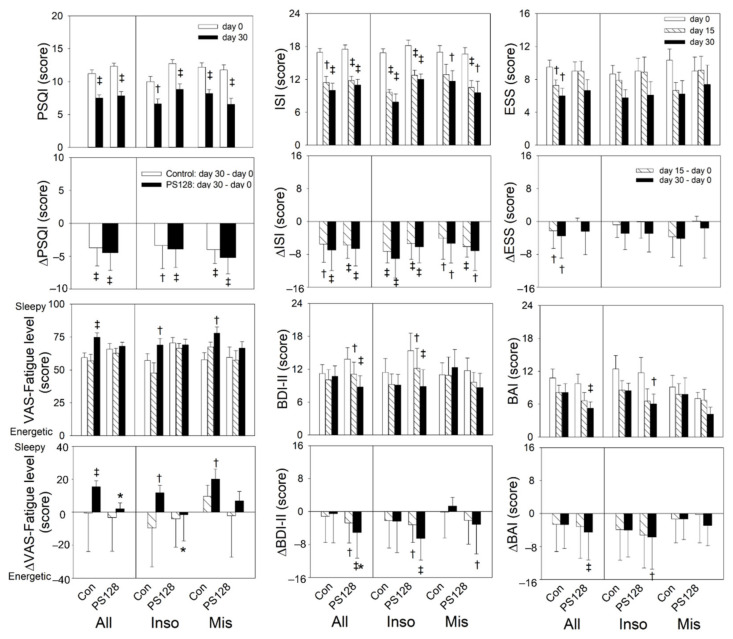
Effect of PS128 and placebo treatment on sleep/mood-related scores after 4 weeks of treatment. Within-groups analysis: both groups showed significant decreases in PSQI, ISI, ESS, BDI-II, and BAI scores compared to baseline. There was a significant decrease between groups in BDI-II and VAS fatigue level before sleep. † *p* < 0.05, ‡ *p* < 0.01 vs. baseline using repeat measurement ANOVA. * *p* < 0.05 vs. the control group by generalized estimating equations. Values are presented as mean ± SEM. All: Con, *n* = 19, PS128, *n* = 21; Inso: Con, *n* = 9, PS128, *n* = 12; Mis: Con, *n* = 10, PS128, *n* = 9. Con, control; Inso, insomniac; Mis, misperception; BMI, body mass index; PSQI, Pittsburgh Sleep Quality Index; ISI, Insomnia Severity Index; ESS, Epworth Sleepiness Scale; BDI-II, Beck Depression Inventory-II; BAI, Beck Anxiety Inventory; VAS, Visual Analogue Scale; WASO, wake after sleep onset; SE, sleep efficiency.

The BDI-II and BAI scores of the PS128 group decreased significantly from baseline after taking capsules for 30 days, and when compared with the control group, the test group showed a significant reduction in BDI-II scores. No difference was observed between the placebo and PS128 groups regarding PSQI, ISI, ESS, and BAI scores (Figure 3, Table S1).

### 3.2. Effects of PS128 on Sleep EEG

**Figure 4:**
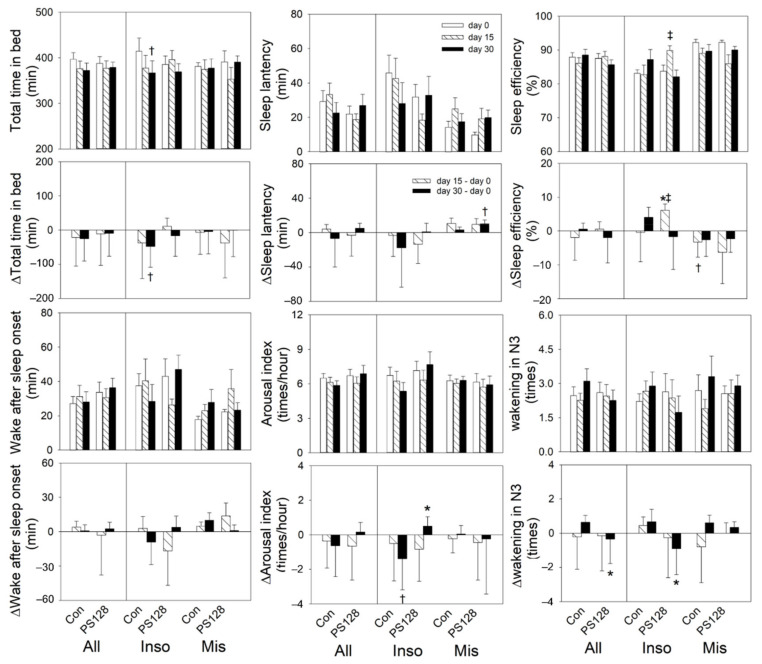
Effects of PS128 on sleep EEG. On day 30, the PS128 group showed significant decreases in awakenings in N3 compared to the control group. † *p* < 0.05, ‡ *p* < 0.01 vs. baseline by repeat measurement ANOVA. * *p* < 0.05 vs. the control group by generalized estimating equations. Values are presented as mean ± SEM. All: Con, *n* = 19, PS128, *n* = 21; Inso: Con, *n* = 9, PS128, *n* = 12; Mis: Con, *n* = 10, PS128, *n* = 9. Con, control; Inso, insomniac; Mis, misperception; N3, non-rapid eye movement sleep stage 3.

**Figure 5:**
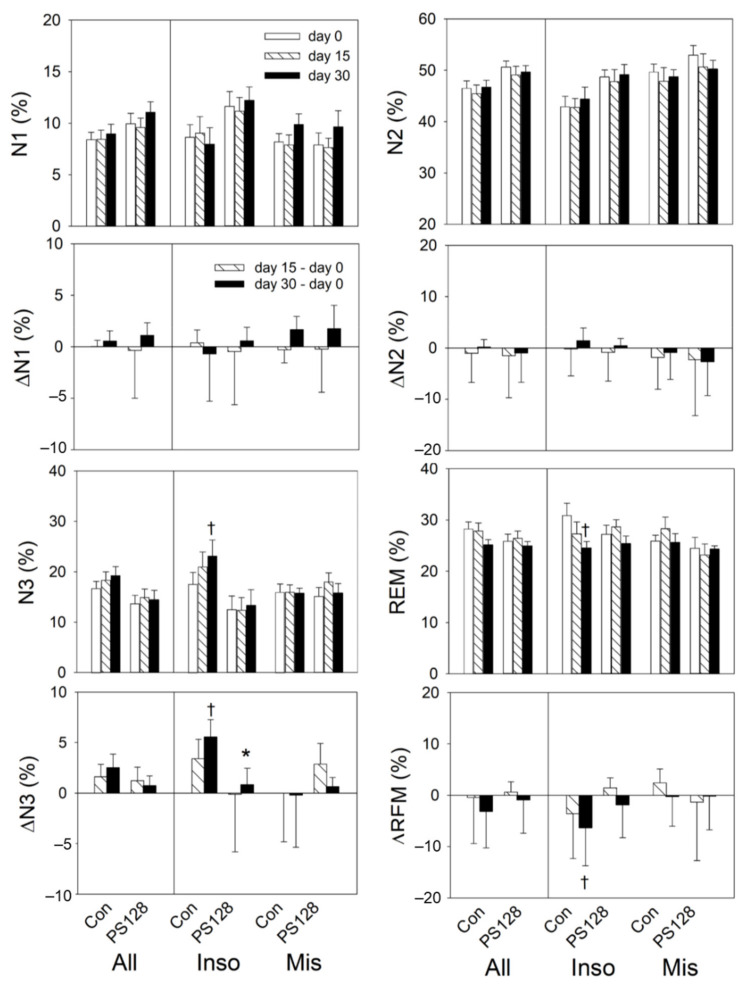
Effects of PS128 on sleep stages. Changes in N1%, N2%, N3%, and REM% on day 0, day 15, and day 30. † *p* < 0.05 vs. baseline by repeated measurement ANOVA. * *p* < 0.05 vs. the control group by generalized estimating equations. Values are presented as mean ± SEM. All: Con, *n* = 19, PS128, *n* = 21; Inso: Con, *n* = 9, PS128, *n* = 12; Mis: Con, *n* = 10, PS128, *n* = 9. N1, non-rapid eye movement sleep (NREM) stage 1; N2, NREM stage 2; N3, NREM stage 3; REM, rapid eye movement sleep. Con, control; Inso, insomniac; Mis, misperception.

Comparing day 30 to baseline, both All and Inso PS128 participants awoke significantly fewer times during N3, compared with those taking a placebo (Figure 4). Within the control group, Inso participants showed significantly decreased total time in bed and REM%, and increased N3% compared to baseline. Between groups, N3% of the control group was significantly higher than that of the PS128 group (Figure 4 and Figure 5, Table S2).

We analyzed the relative values of beta wave, alpha wave, theta wave, and delta wave during overall sleep, but there was no significant difference within or between the PS128 and placebo groups and between the Inso and Mis groups. We subsequently compared brainwaves between the two groups in more detail, by dividing overall sleep into N1, N2, N3, and REM. During N1, the theta power % of the PS128 group on day 15 was significantly decreased (*p* < 0.05 versus control group), and delta power % was greatly increased; however, this change did not reach statistical significance (*p* = 0.076 versus control group). Furthermore, there was no significant difference in N2, N3, and REM. On day 15 and day 30, the beta power %, alpha power %, and theta power % of the PS128 group were lower than those of the control group, whereas the delta power % was higher. In REM, delta power % of the PS128 group on day 15 was a borderline significant increase (Table S3).

### 3.3. Effects of PS128 on HRV

At baseline, the TP and LF values of the two groups showed significant differences. At the end of the study, no significant differences were found between the control and PS128 groups for TP, HF, LF, and LF% (Table S4).

### 3.4. Correlation between Changes in Objective Parameters and BDI-II Scores

**Figure 6:**
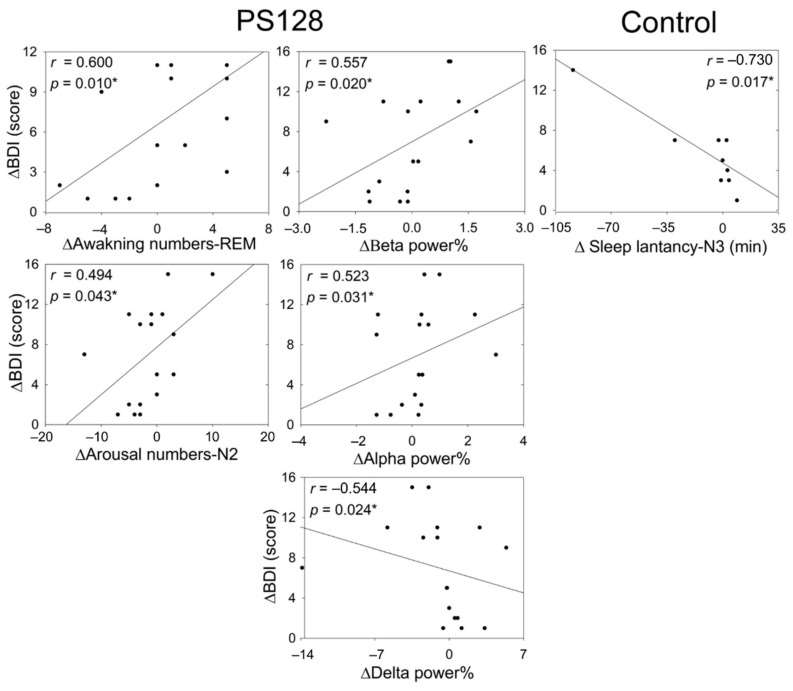
Two dimensional scatter plots displaying the relationship between the change in BDI-II scores and the change in objective parameters from baseline to 4 weeks of treatment. The figure shows those parameters that are correlated to the △BDI. Control, *n* = 10; PS128, *n* = 17. * *p* < 0.05, Spearman rank correlation analysis. BDI-II, Beck Depression Inventory-II; REM, rapid eye movement stage; N2, non-REM (NREM) stage 2; N3, NREM stage 3; △ in scores as the day 0 score minus the day 30 score.

When determining correlations involving BDI-II scores, we calculated the difference (△) in scores as the score on day 0 minus the score on day 30. The BDI-II score was improved if it was greater than zero (△BDI-II > 0). △BDI-II in the PS128 group was positively correlated with the △awakening numbers-REM, △arousal numbers-N2, △beta power %, and △alpha power %. BDI scores in the PS128 group were negatively correlated with △delta power%. In the control group, △BDI-II scores were only negatively correlated with △sleep latency-N3 (Figure 6).

## 4. Discussion

This pilot study examined whether the use of PS128 could reduce the severity of anxiety and depression symptoms, adjust ANS function, and improve the sleep quality of self-reported insomniacs. The results showed that participants in the PS128 group experienced fewer depressive symptoms and fatigue, less frequent awakening and arousal, and decreased high-frequency brain wave activity. The objective parameters indicated more stable sleep in the PS128 group than in the control group. These findings are similar to Armitage’s results [46], in which insomniacs reporting poor sleep were more depressed and had higher alpha wave levels than healthy participants. Those experiencing less stress also had higher delta wave levels. Moreover, BDI-II and BAI scores in the PS128 group improved throughout the study period, suggesting prolonged amelioration of depressive symptoms and anxiety with regular consumption of PS128.

PS128 as a psychobiotic has shown some beneficial effects on mental disorders, such as the autism spectrum disorder (ASD). The probiotic product has been reported to ameliorate anxiety, hyperactivity, impulse, and opposition/defiance behaviors [47], and has more positive effects in children than adolescents with ASD [48]. Combined with oxytocin treatment, PS128 increases ASD patients’ favorable gut microbiome network hubs [49]. Furthermore, it may have a potential benefit for mood disorders, as the main finding of this study suggested that PS128 may be able to reduce depressive symptoms in insomniacs. This is consistent with previous studies on PS128 [31,50]. Although the study did not test biochemical markers, a previous study on PS128 found that its use can increase dopamine and serotonin levels in the brain of mice. These two neurotransmitters are affected by common antidepressant drugs [51], and are classified as excitatory neurotransmitters in the field of sleep medicine. They may inhibit GABA neurons and activate the cortex, causing sleep disturbance [52]. However, the specific role of serotonin on sleep is still unclear. Other research has found contrasting results and showed that a stimulated serotonergic system can promote the onset of sleep in zebrafish and mice [53]. In addition, it may turn into melatonin at night to help shorten sleep latency [54]. Hence, the time of day at which PS128 is taken may be crucial to its effect on sleep quality. In this study, the VAS fatigue level before sleep indicated that the control group felt significantly more tired on day 30 than on day 0, and that there was a significant difference between the two groups. Normally, increased levels of dopamine within the brain promote alertness and sleep disturbances. Alternatively, dopamine deficiency results in fatigue and demotivation. However, a literature review [55] has shown that administering a low dose of a D-2 dopamine receptor agonist will produce a sedative effect, reduce wakefulness, and increase slow-wave and REM sleep. A large dose, on the other hand, induces the opposite effect. As mentioned previously, preclinical studies have shown that PS128 adjusts brain dopamine levels and enhances exercise performance in triathletes [56]. By taking PS128, participants in the intervention group may have avoided fatigue, as the psychobiotic modulated their dopamine levels. However, we were not able to measure brain dopamine levels in human subjects, so we could not determine whether PS128′s beneficial effects on mental health were due to dopamine modulation by the gastrointestinal microbiota.

Compared to the control group, no sleep efficacy of PS128 could be found in the miniature-PSG report, which included some commonly used indices to determine sleep quality, such as SOL, SE, or duration of sleep stages, with the exception of the numbers of wakening in N3. However, PS128 may decrease cortical excitation states. Compared to the control group, delta power percentage was higher in the PS128 group during each stage of sleep, while beta, alpha, and theta power percentages were typically lower during all stages except N1. During N1, especially on day 15, theta wave percentage decreased and delta wave percentage increased in the PS128 compared to the control group. This suggests that PS128 may play a role in changing theta waves into delta waves to promote sleep quality in N1. The same result was seen during REM. The PS128 group had a lower theta wave percentage and a higher delta wave percentage. Previous studies [57,58] indicated that one function of REM is emotional memory storage. Memories that have been temporarily stored in the hippocampus are transferred during REM into the neocortex. This process creates theta waves and may explain the mood-improving effect seen when taking PS128.

The microbiome is unique to each individual. Specific probiotics may affect each person differently. A study investigating the effects of *Lactobacillus casei* Shirota on 132 subjects showed that probiotics only affect those whose mood was initially poor [59]. Thus, we analyzed the correlation between changes in objective parameters and BDI-II scores. We found that when depressive symptoms were alleviated in the PS128 group, beta power percentage and alpha power percentage were reduced, while delta power percentage was increased. In other words, those who respond to PS128 may have less cortical excitation and deep sleep. However, the mechanism by which probiotics affect brainwaves is still unknown. These changes were not observed in the control group. It seems that an outlier among an already small group of trial subjects may have skewed the results.

In addition, there was no significant change between groups or between baseline and treatment in HRV analysis. According to the HPA theory, stress and mood can activate the HPA, release stress hormones, and increase sympathetic activity. During sleep, HF will increase and LF% will decrease. The HRV results suggested that PS128 may not affect HPA and ANS functions.

Both groups had significantly decreased PSQI and ISI scores, and the placebo effect might partially explain this result. Probiotic products are commonly accepted in Taiwan, and people believe that probiotics can improve their health. A previous study [60] using magnetic resonance imaging to test the probiotic effect on mood showed that even the placebo group displayed a noticeable change in the image. As for the measurements, PSQI, ISI, and ESS are some frequently used outcomes in clinical trials, and have certain degrees of reliability and validity [61]. Nevertheless, in the present study, the questionnaires used were all based on self-report, and such measures might not be optimal. The accuracy of self-report measures has long been criticized by psychologists and psychiatrists. Self-reports can easily be subject to context, rapport, and memory bias, etc. Most importantly, the changes in scores cannot be accurately quantified from one individual to another [62]. Another relevant finding of the study is that while PSQI and ISI showed significant changes compared to the baseline, the EEG sleep data did not comply with these questionnaires. This discrepancy was consistent with previous studies [63]. Due to the reasons above, we used the EEG data as a primary outcome and the questionnaires as a secondary outcome.

This study has several limitations. First, intestinal microbiome analysis was not carried out, so we could not confirm whether the probiotic formulation effectively colonized the gut. Internal conditions could cause PS128 to lose probiotic activity. Second, the insufficient sample size might not be able to provide strong statistical evidence to explain the results. The present study consisted of a double-blind randomized design, but the lack of a cross-over design could be a serious limitation when the sample size was so limited. Consequently, a larger sample size is needed for further studies. Third, the results may not be generalizable to other populations because of the small sample size, only recruiting healthy young participants, or the non-response bias resulting from seventy-seven participants’ not replying to our email to confirm enrolment in the study. Fourth, the EEG analysis improperly included sigma activity (12–15 Hz), which mostly corresponds to sleep spindles within the alpha and beta power. Fifth, the diet may be a limitation. We did not restrict or record the diet of the participants throughout the intervention. We only advised the subjects not to consume caffeine on the day of the miniature-PSG measurement. Finally, participants were instructed to take the capsule after dinner, and the timing of this varied among the participates. So far, we still do not know whether the PS128 needs time to show its effect on the body. However, this study has its advantages. The study was a double-blind trial that prevented researchers from making the wrong conclusions, as the questionnaires showed significant changes in both the PS128 and the control group. In addition, few psychobiotic studies use EEG to measure insomniacs’ brainwaves during sleep. Moreover, we used EEG twice during the baseline to minimize the first-night effect.

## 5. Conclusions

Generally, the two groups showed similar results, though some differences were found. Our findings suggested that daily administration of PS128 may lead to a decrease in depressive symptoms, cortical excitation, and fatigue level and an improvement in the quality of deep sleep. PS128 was not found to have a significant effect on ANS function. Further studies with larger sample sizes are needed to clarify the mechanisms underlying the effects of PS128 in alleviating depressive symptoms and cortical excitation, and to determine whether the timing of PS128 intake affects objective sleep quality.

## Figures and Tables

**Table 1:** Baseline demographic data (mean ± SE).

	Control (*n* = 19)			PS128 (*n* = 21)			*p* Value *		
	All	Inso	Mis	All	Inso	Mis	All	Inso	Mis
Male/female	8/11	2/7	6/4	5/16	2/10	3/6			
Age (year)	25.47 ± 4.64	25.11 ± 5.49	25.8 ± 4.02	26.43 ± 5.95	28.58 ± 6.50	25.1 ± 6.05	0.58	0.21	0.23
BMI (kg/m^2^)	22.31 ± 2.37	21.76 ± 2.76	22.81 ± 1.97	21.60 ± 1.80	22.40 ± 1.57	20.89 ± 1.86	0.29	0.55	0.01 *
PSQI	11.26 ± 2.33	10.22 ± 2.22	12.20 ± 2.10	12.33 ± 2.20	12.75 ± 2.05	11.78 ± 2.87	0.14	0.01 *	0.69
ISI	16.74 ± 3.03	16.44 ± 2.30	17 ± 3.68	17.52 ± 3.49	18.17 ± 3.54	16.67 ± 3.43	0.45	0.22	0.84
BDI-II	11.21 ± 6.69	11.44 ± 7.59	11 ± 6.18	13.81 ± 9.64	15 ± 11.17	13.44 ± 7.89	0.33	0.55	0.65
BAI	10.32 ± 6.98	12.44 ± 7.28	8.4 ± 6.47	9.81 ± 7.93	10.75 ± 9.46	8.56 ± 5.59	0.83	0.70	0.88
ESS	9.63 ± 3.59	8.67 ± 3.12	10.5 ± 3.92	9.05 ± 4.75	9.25 ± 5.03	8.78 ± 4.63	0.67	0.76	0.39
MEQ	44.37 ± 10.51	47.56 ± 6.39	41.5 ± 12.86	46.52 ± 13.50	45.08 ± 13.62	48.44 ± 13.90	0.58	0.62	0.27
STAI	48.39 ± 10.45	50.38 ± 10.76	46.8 ± 10.49	48.65 ± 12.17	46.33 ± 13.77	52.13 ± 9.02	0.94	0.49	0.27
SL (min)	29.29 ± 27.20	45.83 ± 30.95	14.40 ± 10.41	22.43 ± 20.70	31.98 ± 22.98	9.70 ± 5.01	0.37	0.50	0.23
WASO (min)	27.23 ± 17.66	37.52 ± 21.00	17.96 ± 5.48	36.04 ± 28.35	46.44 ± 34.23	22.18 ± 4.48	0.25	0.88	0.09
SE (%)	87.95 ± 5.52	83.16 ± 3.15	92.27 ± 2.89	87.23 ± 6.38	83.46 ± 5.90	92.27 ± 2.00	0.71	0.25	1.00

* *p* < 0.05 compared with control group by *t*-test. Inso, insomnia; Mis, misperception; BMI, body mass index; PSQI, Pittsburgh Sleep Quality Index; ISI, Insomnia Severity Index; BDI-II, Beck Depression Inventory-II; BAI, Beck Anxiety Inventory; ESS, Epworth Sleepiness Scale; MEQ, Morningness-Eveningness Questionnaire; STAI, State-Trait Anxiety Inventory State; WASO, wake after sleep onset; SE, sleep efficiency.

## Data Availability

The data presented in this study are available on request from the corresponding author.

## Supplementary Materials

The following are available online at https://www.mdpi.com/article/10.3390/nu13082820/s1. Table S1: the parameters of subjective questionnaires by general estimating equations analysis. Table S2: objective sleep parameters by general estimating equations analysis. Table S3: brainwaves outcomes during N1, N2, N3, and REM sleep by general estimating equations analysis. Table S4: heart rate variability outcomes for both study groups during sleep by general estimating equations analysis.
