# Supplementary material for: Effects of Lactobacillus plantarum PS128 on Depressive Symptoms and Sleep Quality in Self-Reported Insomniacs: A Randomized, Double-Blind, Placebo-Controlled Pilot Trial
Source: Nutrients. 2021 Aug 17;13(8):2820. doi: 10.3390/nu13082820 (PMC8402034; doi:10.3390/nu13082820)
Supplement: Supplementary file 1 [file nutrients-13-02820-s001.zip › nutrients-1314607-supplementary.pdf]

Supplementary Materials

Effects of *Lactobacillus plantarum* PS128 on Depressive Symptoms and Sleep Quality in Self-Reported Insomniacs: A Randomized, Double-Blind, Placebo-Controlled Pilot Trial

Yu-Ting Ho [1,2,†], Ying-Chieh Tsai [3,†], Terry B. J. Kuo [1,2,4,5,6] and Cheryl C. H. Yang [1,2,4,5,*]

[1] Institute of Brain Science, National Yang Ming Chiao Tung University, Taipei 11211, Taiwan; mascottofu@gmail.com (Y.-T.H.); tbjkuo@ym.edu.tw (T.B.J.K.)
[2] Sleep Research Center, National Yang Ming Chiao Tung University, Taipei 11211, Taiwan
[3] Institute of Biochemistry and Molecular Biology, National Yang Ming Chiao Tung University, Taipei 11211, Taiwan; tsaiyc@ym.edu.tw
[4] Brain Research Center, National Yang Ming Chiao Tung University, Taipei 11211, Taiwan
[5] Department of Education and Research, Taipei City Hospital, Taipei 103212, Taiwan
[6] Clinical Research Center, Taoyuan Psychiatric Center Ministry of Health and Welfare, Taoyuan 33058, Taiwan
* Correspondence: cchyang@ym.edu.tw; Tel.: +886-2-2826-7058
† These authors contributed equally to this study.

Table S1. The parameters of subjective questionnaires by general estimating equations analysis.

				Wald (95% Cl)			
		β	*S.E.*	lower	upper	Wald X^2	*p*
PSQI	residual	9.647	1.1729	7.348	11.946	67.642	0
	day30	-0.719	0.8442	-2.374	0.935	0.726	0.394
ISI	residual	18.065	1.7978	14.541	21.588	100.972	0
	day15	-0.273	1.2238	-2.672	2.125	0.05	0.823
	day30	0.275	1.4522	-2.571	3.122	0.036	0.85
ESS	residual	10.007	3.1821	3.77	16.243	9.89	0.002
	day15	2.323	1.2784	-0.183	4.828	3.301	**0.069**
	day30	1.333	1.7758	-2.147	4.814	0.564	0.453
VAS	residual	50.009	10.62	29.194	70.824	22.174	0
-emotion before sleep	day15	-3.859	6.3932	-16.389	8.672	0.364	0.546
	day30	-0.421	5.4103	-11.025	10.183	0.006	0.938
VAS	residual	51.197	10.6017	30.418	71.976	23.321	0

-fatigued before sleep	day15	-1.905	6.9552	-15.537	11.727	0.075	0.784
	day30	-13.378	4.9958	-23.17	-3.587	7.171	**0.007***
VAS	residual	26.866	11.6233	4.085	49.647	5.343	0.021
-sleep quality	day15	9.491	9.0391	-8.225	27.208	1.103	0.294
	day30	-3.579	8.9705	-21.161	14.003	0.159	0.69
VAS	residual	26.866	11.6233	4.085	49.647	5.343	0.021
-fatigued after sleep	day15	9.491	9.0391	-8.225	27.208	1.103	0.294
	day30	-3.579	8.9705	-21.161	14.003	0.159	0.69
BDI-II	residual	12.572	6.9911	-1.13	26.275	3.234	0.072
	day15	-1.682	1.783	-5.176	1.813	0.89	0.346
	day30	-4.832	2.0199	-8.791	-0.873	5.723	**0.017***
BAI	residual	14.378	4.056	6.428	22.328	12.566	0
	day15	-0.407	2.252	-4.82	4.007	0.033	0.857
	day30	-1.727	1.8908	-5.433	1.979	0.834	0.361

[a] The reference group is control group*baseline.

[b] Adjusted for age and sex

[c] *PSQI* Pittsburgh Sleep Quality Index; *ISI* Insomnia Severity Index; *ESS* Epworth Sleepiness Scale; *VAS* Visual Analogue Scale; *BDI-II* Beck Depression Inventory-II; *BAI* Beck Anxiety Inventory; β regression coefficients; *S.E* standard error; *CI* confidence interval.

[d] $^{*}p < 0.05$, $^{**}p < 0.01$ vs. the control group. Boldface letters for p indicate borderline significance.

Table S2. Objective sleep parameters by general estimating equations analysis.

				Wald (95% Cl)			
		β	*S.E.*	lower	upper	Wald X^2	*p*
Total time in bed (min)	residual	485.694	37.9389	411.335	560.053	163.891	0
	day15	-9.486	28.5458	-65.435	46.462	0.11	0.74
	day30	21.194	24.9605	-27.727	70.116	0.721	0.396
Sleep period (min)	residual	419.371	37.9518	344.987	493.755	122.104	0
	day15	-1.942	27.7203	-56.273	52.389	0.005	0.944
	day30	10.963	21.9319	-32.023	53.949	0.25	0.617
Wake after sleep onset (min)	residual	-6.32	17.2868	-40.201	27.562	0.134	0.715
	day15	-8.759	9.1184	-26.631	9.113	0.923	0.337
	day30	-0.85	7.6513	-15.847	14.146	0.012	0.912
Total sleep time (min)	residual	427.576	32.9515	362.992	492.16	168.375	0
	day15	6.373	27.5483	-47.621	60.366	0.054	0.817
	day30	11.814	20.528	-28.421	52.048	0.331	0.565
Sleep efficiency (%)	residual	87.059	4.0195	79.181	94.937	469.116	0
	day15	2.748	2.5785	-2.305	7.802	1.136	0.286
	day30	-1.896	2.3861	-6.572	2.781	0.631	0.427
N1%	residual	-0.102	2.3496	-4.707	4.503	0.002	0.965

	day15	-0.596	1.1713	-2.891	1.7	0.259	0.611
	day30	0.205	1.532	-2.798	3.208	0.018	0.894
N2%	residual	40.038	3.7796	32.63	47.446	112.215	0
	day15	0.022	2.2269	-4.343	4.386	0	0.992
	day30	-0.399	1.9791	-4.278	3.48	0.041	0.84
N3%	residual	34.113	4.4346	25.421	42.804	59.173	0
	day15	-0.599	1.7796	-4.087	2.889	0.113	0.737
	day30	-2.208	1.6264	-5.395	0.98	1.843	0.175
REM%	residual	27.339	3.3318	20.808	33.869	67.327	0
	day15	0.907	2.7611	-4.505	6.318	0.108	0.743
	day30	2.226	2.0851	-1.86	6.313	1.14	0.286
Awakening index	residual	2.302	1.2766	-0.2	4.804	3.252	0.071
	day15	-0.116	0.4556	-1.009	0.777	0.064	0.8
	day30	0.042	0.4979	-0.934	1.017	0.007	0.933
Number of awakenings	residual	-3.399	2.8186	-8.923	2.126	1.454	0.228
from N1	day15	-2.524	1.663	-5.784	0.735	2.304	0.129
	day30	0.471	1.7694	-2.997	3.939	0.071	0.79
Number of awakenings	residual	8.496	3.9228	0.807	16.184	4.69	0.03
from N2	day15	1.263	2.4817	-3.601	6.127	0.259	0.611

	day30	-0.18	2.1424	-4.38	4.019	0.007	0.933
Number of awakenings	residual	5.471	0.8596	3.787	7.156	40.516	0
from N3	day15	0.021	0.6131	-1.18	1.223	0.001	0.972
	day30	-1.032	0.5031	-2.018	-0.046	4.204	**0.04***
Number of awakenings	residual	5.82	3.5409	-1.12	12.76	2.702	0.1
from REM	day15	1.614	1.6482	-1.617	4.844	0.959	0.328
	day30	1.717	1.3848	-0.997	4.431	1.537	0.215
Sleep latency to N1 (min)	residual	68.604	13.5371	42.072	95.136	25.683	0
	day15	-7.835	7.2988	-22.14	6.471	1.152	0.283
	day30	10.21	9.2983	-8.014	28.434	1.206	0.272
Sleep latency to N2 (min)	residual	73.028	13.6679	46.239	99.816	28.548	0
	day15	-6.127	8.3709	-22.534	10.279	0.536	0.464
	day30	11.767	10.0121	-7.857	31.39	1.381	0.24
Sleep latency to N3 (min)	residual	-5.515	14.1343	-33.218	22.188	0.152	0.696
	day15	-2.073	5.0736	-12.017	7.871	0.167	0.683
	day30	4.471	8.304	-11.805	20.747	0.29	0.59
Sleep latency to REM	residual	58.804	20.2177	19.178	98.43	8.46	0.004
(min)	day15	-8.137	11.179	-30.048	13.773	0.53	0.467
	day30	18.372	12.8398	-6.793	43.538	2.047	0.152

Number of arousals	residual	27.3	13.0735	1.676	52.924	4.361	0.037
	day15	-0.687	4.6157	-9.734	8.36	0.022	0.882
	day30	5.744	4.4651	-3.007	14.496	1.655	0.198
Arousal index	residual	3.797	1.7597	0.348	7.246	4.655	0.031
	day15	-0.327	0.547	-1.399	0.745	0.358	0.55
	day30	0.76	0.6516	-0.517	2.037	1.36	0.244
Number of arousals in N1	residual	0.261	5.7509	-11.011	11.532	0.002	0.964
	day15	-3.582	1.8589	-7.226	0.061	3.713	**0.054**
	day30	2.962	2.4619	-1.863	7.788	1.448	0.229
Number of arousals in N2	residual	14.241	5.6059	3.253	25.228	6.453	0.011
	day15	0.858	2.458	-3.96	5.676	0.122	0.727
	day30	2.278	2.2275	-2.088	6.644	1.046	0.306
Number of arousals in N3	residual	3.44	0.9961	1.488	5.392	11.926	0.001
	day15	0.454	0.5639	-0.652	1.559	0.647	0.421
	day30	0.416	0.6104	-0.78	1.612	0.465	0.495
Number of arousals in	residual	11.206	4.268	2.84	19.571	6.893	0.009
REM	day15	1.797	2.5081	-3.119	6.713	0.513	0.474
	day30	0.516	2.388	-4.164	5.197	0.047	0.829

[a] The reference group is control group*baseline.

[b] Adjusted for age and sex

[c] *REM* Rapid eye movement stage; *N1* non-REM stage 1; *N2* non-REM stage 2; *N3* non-REM stage 3; β regression coefficients; *S.E* standard error; *CI* confidence interval.

[d] *$p < 0.05$, **$p < 0.01$, vs. the control group. Boldface letters for p indicate borderline significance.

Table S3. Brainwaves outcomes during N1, N2, N3, and REM sleep by general estimating equations analysis.

				Wald (95% Cl)			
		β	*S.E.*	lower	upper	Wald X^2	*p*
			**overall brain activity during sleep**				
Beta power (%)	residual	1.65	0.8951	-0.104	3.404	3.398	0.065
	day15	-0.248	0.3966	-1.025	0.53	0.39	0.532
	day30	0.106	0.325	-0.531	0.743	0.106	0.744
Alpha power (%)	residual	4.048	1.4431	1.22	6.877	7.87	0.005
	day15	-0.504	0.3895	-1.268	0.259	1.678	0.195
	day30	-0.042	0.3339	-0.697	0.612	0.016	0.899
Theta power (%)	residual	7.738	1.8673	4.078	11.398	17.172	0
	day15	-0.805	0.7348	-2.245	0.635	1.202	0.273
	day30	-0.575	0.6474	-1.843	0.694	0.788	0.375
Delta power (%)	residual	86.534	3.2258	80.211	92.856	719.622	0
	day15	1.562	1.2714	-0.93	4.054	1.509	0.219
	day30	0.511	1.1346	-1.713	2.735	0.203	0.652
			**N1**				
Beta power (%)	residual	3.967	1.3197	1.38	6.553	9.036	0.003
	day15	-0.107	0.5885	-1.26	1.047	0.033	0.856

	day30	0.032	0.5455	-1.037	1.101	0.003	0.953
Alpha power (%)	residual	5.122	1.4638	2.253	7.991	12.244	0
	day15	-0.519	0.5177	-1.533	0.496	1.004	0.316
	day30	-0.033	0.4486	-0.912	0.846	0.005	0.941
Theta power (%)	residual	10.49	2.6545	5.287	15.693	15.616	0
	day15	-2.274	0.959	-4.154	-0.395	5.625	**0.018***
	day30	-1.073	0.8912	-2.82	0.674	1.449	0.229
Delta power (%)	residual	80.426	3.9991	72.588	88.265	404.459	0
	day15	2.902	1.6376	-0.308	6.111	3.14	**0.076**
	day30	1.074	1.5851	-2.033	4.181	0.459	0.498
			**N2**				
Beta power (%)	residual	2.462	0.9152	0.668	4.256	7.235	0.007
	day15	-0.023	0.4323	-0.871	0.824	0.003	0.957
	day30	-0.083	0.3595	-0.787	0.622	0.053	0.818
Alpha power (%)	residual	5.997	1.8663	2.339	9.655	10.325	0.001
	day15	-0.297	0.3915	-1.064	0.471	0.574	0.449
	day30	-0.449	0.3628	-1.161	0.262	1.534	0.215
Theta power (%)	residual	8.169	1.9443	4.358	11.98	17.652	0
	day15	-0.749	0.739	-2.197	0.699	1.027	0.311

	day30	-1.029	0.6653	-2.333	0.275	2.394	0.122
Delta power (%)	residual	83.358	3.3688	76.755	89.961	612.257	0
	day15	1.077	1.3421	-1.554	3.707	0.644	0.422
	day30	1.562	1.1524	-0.697	3.82	1.836	0.175
			**N3**				
Beta power (%)	residual	0.194	0.4479	-0.684	1.072	0.187	0.665
	day15	-0.076	0.1323	-0.335	0.184	0.328	0.567
	day30	-0.024	0.1238	-0.267	0.218	0.038	0.845
Alpha power (%)	residual	-0.944	1.706	-4.288	2.4	0.306	0.58
	day15	-0.344	0.2143	-0.764	0.076	2.581	0.108
	day30	-0.171	0.2518	-0.664	0.323	0.461	0.497
Theta power (%)	residual	1.06	1.0989	-1.094	3.214	0.931	0.335
	day15	-0.438	0.4188	-1.258	0.383	1.091	0.296
	day30	-0.162	0.3304	-0.81	0.486	0.24	0.624
Delta power (%)	residual	99.697	2.219	95.348	104.046	2018.571	0
	day15	0.858	0.6169	-0.351	2.067	1.935	0.164
	day30	0.357	0.5759	-0.772	1.486	0.384	0.535
			**REM**				
Beta power (%)	residual	7.375	1.2813	4.863	9.886	33.127	0

	day15	-0.953	0.5952	-2.12	0.213	2.566	0.109
	day30	-0.26	0.4843	-1.21	0.689	0.289	0.591
Alpha power (%)	residual	9.796	1.3892	7.073	12.518	49.719	0
	day15	-0.649	0.5719	-1.77	0.472	1.287	0.257
	day30	-0.23	0.6368	-1.478	1.018	0.131	0.718
Theta power (%)	residual	19.137	2.1394	14.944	23.33	80.012	0
	day15	-1.67	0.9513	-3.534	0.195	3.081	**0.079**
	day30	-1.201	0.7949	-2.759	0.357	2.283	0.131
Delta power (%)	residual	63.43	3.2135	57.131	69.728	389.604	0
	day15	3.296	1.7733	-0.179	6.772	3.455	**0.063**
	day30	1.692	1.4691	-1.188	4.571	1.326	0.25

[a] The reference group is control group*baseline.

[b] Adjusted for age and sex

[c] *REM* rapid eye movement stage; *N1* non-REM stage 1; *N2* non-REM stage 2; *N3* non-REM stage 3; *β* regression coefficients; *S.E* standard error; *CI* confidence interval.

[d] *$p < 0.05$, **$p < 0.01$ vs. the control group. Boldface letter for *p* means borderline significance.

Table S4. Heart rate variability outcomes for both study groups during sleep by general estimating equations analysis.

				Wald (95% Cl)			
		β	*S.E.*	lower	upper	Wald X^2	*p*
RR	residual	989.636	69.0609	854.279	1124.993	205.346	0
	day15	-28.702	20.5714	-69.021	11.618	1.947	0.163
	day30	14.743	24.8619	-33.985	63.472	0.352	0.553
TP	residual	7.847	0.4404	6.984	8.711	317.495	0
	day15	-0.118	0.1267	-0.366	0.13	0.865	0.352
	day30	0.14	0.1211	-0.097	0.378	1.345	0.246
HF	residual	6.738	0.546	5.668	7.809	152.288	0
	day15	-0.243	0.1501	-0.537	0.051	2.619	0.106
	day30	0.037	0.1503	-0.258	0.332	0.06	0.806
LF	residual	6.807	0.4359	5.953	7.662	243.916	0
	day15	-0.054	0.1386	-0.326	0.217	0.155	0.694
	day30	0.19	0.1254	-0.056	0.436	2.3	0.129
LF/HF	residual	0.036	0.355	-0.66	0.732	0.01	0.92
	day15	0.186	0.0972	-0.005	0.376	3.647	**0.056**
	day30	0.153	0.1059	-0.054	0.361	2.09	0.148
LF%	residual	51.509	7.1165	37.561	65.457	52.387	0

	day15	3.355	1.9948	-0.555	7.264	2.828	0.093
	day30	3.036	2.1823	-1.242	7.313	1.935	0.164

[a] The reference group is control group*baseline

[b] Adjusted for age and sex

[c] *RR* R-R interval; *TP* total power; *HF* high frequency; *LF* low frequency; *LF*% normalised low frequency power; *β* regression coefficients; *S.E* standard error; *CI* confidence interval.

[d] $*p < 0.05$, $**p < 0.01$ vs. the control group. Boldface letters for *p* indicate borderline significance.
